# Timing for intracoronary administration of bone marrow mononuclear cells after acute ST-elevation myocardial infarction: a pilot study

**DOI:** 10.1186/s13287-015-0102-5

**Published:** 2015-05-29

**Authors:** Rongchong Huang, Kang Yao, Aijun Sun, Juying Qian, Lei Ge, Yiqi Zhang, Yuhong Niu, Keqiang Wang, Yunzeng Zou, Junbo Ge

**Affiliations:** The First Affiliated Hospital of Dalian Medical University, 222 Zhongshan Road, Dalian, 116011 China; Shanghai Institute of Cardiovascular Diseases, Zhongshan Hospital, Fudan University, 180 Feng Lin Road, Shanghai, 200032 China; Institutes of Biomedical Science, Fudan University, 138 Dong’an Road, Shanghai, 200032 China

## Abstract

**Introduction:**

Most studies on intracoronary bone marrow mononuclear cell transplantation for acute myocardial infarction involve treatment 3–7 days after primary percutaneous coronary intervention (PCI); however, the optimal timing is unknown. The present study assessed the therapeutic effect at different times after ST-elevation myocardial infarction.

**Methods:**

The present trial was not blinded. A total of 104 patients with a first ST-elevation myocardial infarction and a left ventricular ejection fraction below 50 %, who had PCI of the infarct-related artery, were randomly assigned to receive intracoronary infusion of bone marrow mononuclear cells within 24 hours (group A, n = 27), 3 to 7 days after PCI (group B, n = 26), or 7 to 30 days after PCI (group C, n = 26), or to the control group (n = 25), which received saline infusion performed immediately after emergency PCI. All patients in groups A, B and C received an injection of 15 ml cell suspension containing approximately 4.9 × 10^8^ bone marrow mononuclear cells into the infarct-related artery after successful PCI.

**Results:**

Compared to control and group C patients, group A and B patients had a significantly higher absolute increase in left ventricular ejection fraction from baseline to 12 months (change: 3.4 ± 5.7 % in control, 7.9 ± 4.9 % in group A, 6.9 ± 3.9 % in group B, 4.7 ± 3.7 % in group C), a greater decrease in left ventricular end-systolic volumes (change: −6.4 ± 15.9 ml in control, −20.5 ± 13.3 ml in group A, −19.6 ± 11.1 ml in group B, −9.4 ± 16.3 ml in group C), and significantly greater myocardial perfusion (change from baseline: −4.7 ± 5.7 % in control, −7.8 ± 4.5 % in group A, −7.5 ± 2.9 % in group B, −5.0 ± 4.0 % in group C). Group A and B patients had similar beneficial effects on cardiac function (*p* = 0.163) and left ventricular geometry (left ventricular end-distolic volume: *p* = 0.685; left ventricular end-systolic volume: *p* = 0.622) assessed by echocardiography, whereas group C showed similar results to those of the control group. Group B showed more expensive care (*p* < 0.001) and longer hospital stays during the first month after emergency PCI (*p* < 0.001) than group A, with a similar improvement after repeat cardiac catheterization following emergency PCI.

**Conclusion:**

Cell therapy in acute myocardial infarction patients that is given within 24 hours is similar to 3–7 days after the primary PCI.

**Trial registration:**

NCT02425358, registered 30 April 2015

## Introduction

On the basis of experimental studies that bone marrow mononuclear cell (BMC) transfer in the injured tissue can promote regional myocardial perfusion and improved cardiac function, several clinical trials have shown that intracoronary BMC transplantation in acute myocardial infarction (AMI) patients several days after myocardial reperfusion is safe and may enhance the improvement of left ventricular ejection fraction (LVEF) [1–6]. The timing of BMC administration, baseline LVEF, dosage of BMC and other factors have been linked to improvement in LVEF after BMC transplantation. In our previous work, we gave BMCs within 24 hours after emergency percutaneous coronary intervention (PCI) and found that it was safe and effective [7]. In addition, there is another report with a longer time from symptom onset to BMC infusion (2–4 weeks) which also appeared effective [4]. The timing of intracoronary stem cell administration may have a critical effect on cell engraftment and may be responsible for the various biological and functional responses to therapy [8, 9]. However, few studies have directly addressed the optimal timing of cell injections. Therefore, in this prospective randomized study, BMCs were given at different times (within 24 hours, 3 to 7 days, or 7 to 30 days after reperfusion) to investigate whether the timing of therapy affects the therapeutic response of AMI patients.

## Methods

### Study protocols

Our institutional ethics committee (medical ethics committee of Zhongshan Hospital, Fudan University) approved the study, and all patients gave their written informed consent. The study was performed according to the principles of the Declaration of Helsinki.

Patients with AMI who were admitted to Zhongshan Hospital, Fudan University, China, were included. The inclusion criteria were: aged 18 to 75 years; a history of first acute ST-elevation myocardial infarction (STEMI); treatment with PCI 2 to 12 hours after symptom onset; successful PCI with stent implantation in the culprit lesion of the infarct-related artery (IRA); and an LVEF <50 % on angiography immediately after emergency PCI or rescue PCI. The exclusion criteria were: previous Q-wave myocardial infarction, cardiogenic shock, and severe coexisting conditions such as acute and chronic heart failure, malignant arrhythmia, renal failure and severe bleeding that interfered with the ability of the patient to comply with the protocol. All patients received medication according to current guidelines.

The trial was not blinded. The day of acute PCI was defined as day 0. On day 0, when patients were admitted to the emergency room, they were informed regarding random intracoronary BMC infusion if LVEF was less than 50 % after primary PCI. The informed consent included the background, the purpose, and the procedure of this trial. The risks and potential benefits of BMC collection, preparation and transplantation were described in detail. A minimum follow-up period of 1 year was requested from all patients. The patients had the right to withdraw from the study at any time. Patients who refused to sign the informed consent were excluded.

**Fig. 1:**
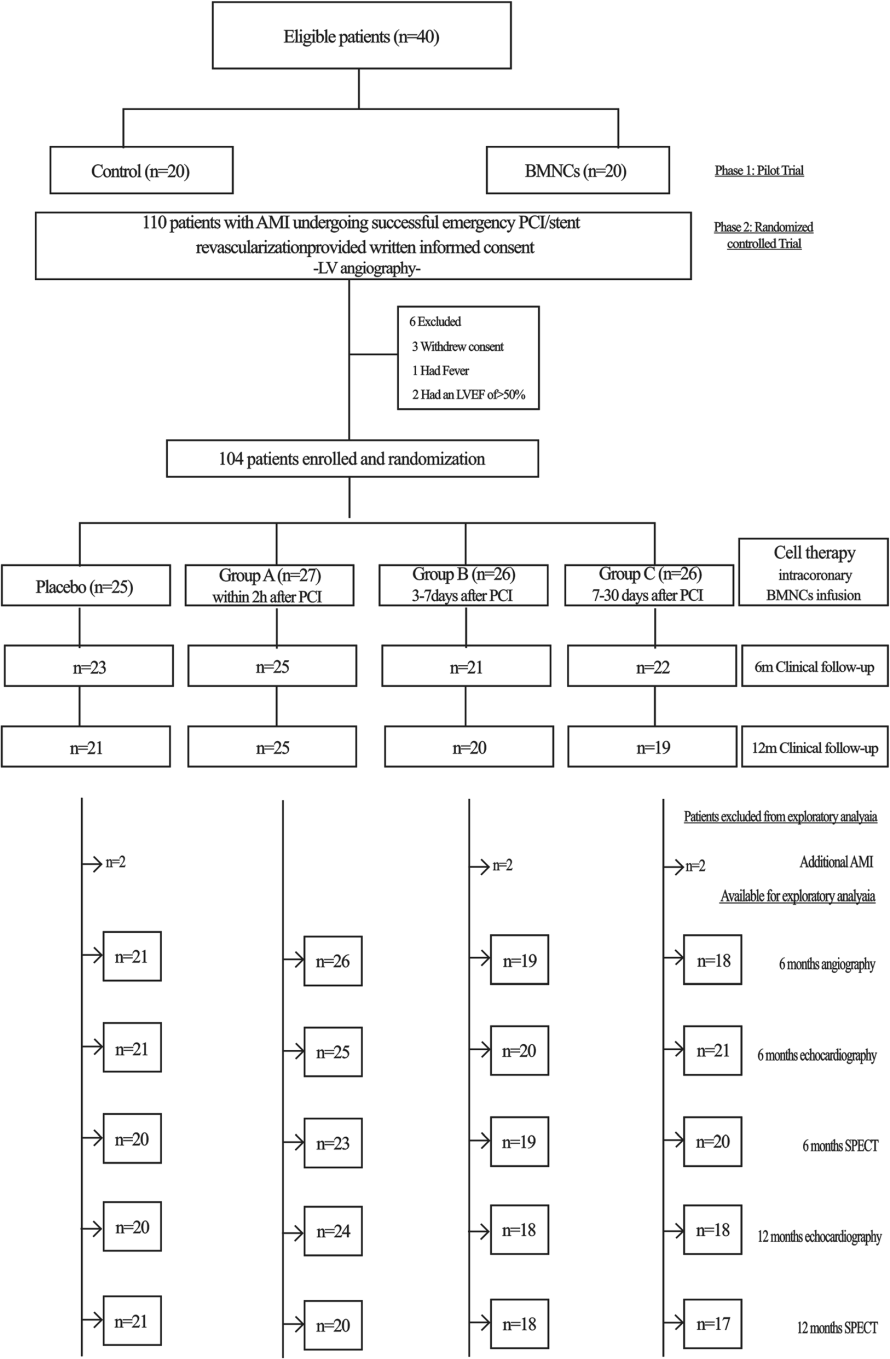
Flow chart outlining the study protocol. A total of 104 acute myocardial infarction (*AMI*) patients were enrolled and randomly assigned to four groups in this trial depending on bone marrow mononuclear cell (*BMNC*) transplantation after primary percutaneous coronary intervention (*PCI*). Before discharge, the patients underwent echocardiography and single photon emission computed tomography (*SPECT*) and the data were collected as baseline. At the 6-month follow-up, patients underwent angiography. The SPECT and echocardiography data were recorded at 6 and 12 months. *Group A* BMNC infusion within 24 hours after PCI; *group B* BMNC infusion at 3–7 days after PCI; *group C* BMNC infusion at 7–30 days after PCI; *LV*, left ventricular; *LVEF* left ventricular ejection fraction

The patients enrolled in this trial were assigned to the following groups: group A, intracoronary infusion of BMC within 24 hours after PCI; group B, intracoronary infusion of BMC 3 to 7 days after PCI; group C, intracoronary infusion of BMC 7 to 30 days after PCI; or the control group (CON), which was given saline infusion immediately after emergency PCI (Fig. 1). After the primary PCI, patients with LVEF ≥50 % were excluded. Finally, there were 27 patients in group A, 26 patients in group B, 26 patients in group C and 25 patients in the CON group.

For patients assigned to receive BMC, a bone marrow sample (95 ± 16 ml) was collected at the designated time points after primary PCI under local anesthesia from both sides of the posterior superior iliac spine. To collect sufficient amounts of bone marrow, several puncture points were required. Patients received an injection of 15 ml cell suspension containing approximately 4.9 × 10^8^ BMCs into the IRA within 3 hours after BMC collection. Patients in group A remained in the cath-lab until the entire procedure, including primary PCI and intracoronary BMC infusion, was completed. However, patients in groups B and C, who underwent a second procedure, to receive BMC transplantation in the cath-lab during the same hospitalization or returned for a second hospitalization. As shown in Fig. 1, after BMC transplantation, all patients underwent clinical examinations, onventional echocardiography, 201Ti single photon emission computed tomography (SPECT), and coronary angiography at the certain time-points. Major adverse cardiac events and restenosis were recorded during the 12 months of follow-up.

### Cell preparation and administration

The BMCs were isolated by Ficoll density gradient centrifugation and were infused into the IRA as described previously [7]. Briefly, BMCs were isolated by Ficoll density gradient centrifugation on Lymphocyte Separation Medium. Three washing steps were performed and cells were resuspended in heparinized saline for use. Viability was tested by Trypan Blue (exclusion method), which showed a viability of more than 99 % cells for each transplant. Repeated microbiological tests of the cell suspensions performed prior to transplantation were negative for each transplant. The levels of CD34+ cells and CD133+ cells were measured.

BMCs were infused into IRA at the site of the previous occlusion. This was accomplished with the use of a microtubular. After positioning of the microtubular into the distal segment vessel of the stent position in the IRA, 15 mL of the whole cell suspension (groups A, B and C) or saline (CON) was slowly administered via microtubular. The administration should be over 10 minutes to prevent back-flow and to prolong cellular contact time for cellular migration into the tissue. After completion of intracoronary cell transplantation, coronary angiography was repeated to ascertain vessel patency and unimpeded flow of contrast material. During the process of intracoronary infusion of BMCs, complications should be watched for, including arrhythmia, bradycardia, sinus arrest or atrial ventricular block, premature ventricular beats, ventricular tachycardia, ventricular fibrillation, and hypotension, and so forth.

### Hemodynamic assessment

LVEF, left ventricular end-diastolic volume (LVEDV), and left ventricular end-systolic volume (LVESV) were measured before discharge, at 6 months, and at 12 months after intracoronary BMC infusion using two-dimensional echocardiography according to Simpson’s method [10]. The measurements were repeated three times, and the average was used for further calculations. All studies were processed and evaluated in the echocardiogram laboratory by experienced operators who were blinded to both the order of the procedures and the assigned therapy.

### 201Tl -SPECT imaging

As shown in Fig. 1, all patients underwent 1-day electrocardio-gated stress Thallium-201 SPECT imaging [11] before discharge, at 6 months, and 12 months after intracoronary BMC infusion. All images were acquired using a tri-head SPECT gamma camera (Philips-IRIX, Philips Medical Systems, Milpitas, CA, USA). The myocardial perfusion defect was automatically calculated using ECTb3.0 software. Data were processed and evaluated in the scintigraphic core laboratory by experienced operators who were blinded to both the order of the procedures and the assigned therapy.

### Statistical analysis

Values for continuous variables that approximated a normal distribution are presented as means ± SD unless otherwise noted. Univariate differences between groups were performed with analysis of variance (ANOVA) for multiple comparisons and Bonferroni’s post test. Statistical comparisons between the initial and follow-up data were performed with paired *t* test. Comparisons of the changes from baseline to 12 months in the control and BMC treatment groups were performed using repeated-measures two-way ANOVA. The ANOVA model included the control versus BMC treatments and baseline versus 12 months as factors, and also included the interaction between the two factors. Categorical variables were analyzed using the chi-square test or Fisher’s exact test, as appropriate. A *p* value of less than 0.05 was considered to indicate statistical significance. All reported *p* values are two-sided. Statistical analyses were performed using STATA software (version 8.0, STATA, U.S.A.).

## Results

### Clinical characteristics

The baseline characteristics of the 104 patients are summarized in Table 1. The average time from AMI to IRA opening was 7 (2–12) hours. As shown in Table 1, the four groups were comparable with respect to gender ratio, age, family history of coronary heart diseases, history of smoking, drinking, diabetes, hypertension, hyperlipidemia, hyperuricemia, interventional therapy, and other variables. The average number of BMCs implanted was (4.9 ± 2.8) × 10^8^. The number of CD34+ cells and CD133+ cells included in the implanted BMCs was (1.4 ± 0.9) × 10^6^ and (3.1 ± 2.2) × 10^5^, respectively. There was no difference in the number of BMC among groups A, B, and C. Cell viability was tested using Trypan Blue (exclusion method); more than 99 % of cells were viable for each transplant.

**Table 1:** Baseline clinical and angiographic characteristics

	CON	Group A	Group B	Group C	*p* value
	(n = 25)	(n = 27)	(n = 26)	(n = 26)	
Age, years	58.8 ± 8.4	60.0 ± 7.0	58.3 ± 9.8	57.3 ± 10.5	0.747
Female, %	12	7	12	8	0.915
Hypertension, %	48	59	50	73	0.490
Hyperlipidemia, %	48	26	65	42	0.281
Diabetes, %	32	31	15	15	0.544
Previous angina, %	24	30	23	31	0.901
Smoking (current or former), %	40	54	62	31	0.130
Family history for CAD, %	28	26	54	46	0.128
CAD (1-/2-/3-vessel disease), n	19/5/1	22/3/2	19/4/3	17/8/1	0.810
Infarct territory (anterior/inferior), %	85/15	70/30	69/27	73/23	0.564
Infarct-related vessel (LAD/RCA/LCX), %	80/16/4	82/11/7	73/23/4	73/19/8	0.911
Previous interventional therapy, n	3	5	2	4	0.638
PCI for additional stenosis in non-infarct-related vessels, n	3	4	3	4	0.970
Time to reperfusion/stent, hours	7.0 ± 2.1	7.0 ± 2.2	5.9 ± 3.5	6.6 ± 3.2	0.766
TIMI flow grade before PCI	0.32 ± 0.69	0.37 ± 0.69	0.31 ± 0.74	0.38 ± 0.80	0.977
Thrombolysis before PCI, n	5	4	3	7	0.702
Drug eluting stent/bare stent/no stent, n	10/15/0	19/8/0	10/14/2	10/14/2	0.766
GPIIb/IIIa inhibitor during acute PCI, %	20	15	12	15	0.876
Intravenous catecholamine, n	2	2	1	1	0.875
CPR during AMI, n	2	1	0	2	0.501
Creatine kinase MB max, U/L	158.6 ± 98.5	169.2 ± 102.0	153.8 ± 74.3	160.1 ± 88.1	0.940
Troponin T max, ng/mL	11.0 ± 10.3	10.0 ± 7.5	9.3 ± 9.0	8.9 ± 6.7	0.818
CRP max, mg/dl	12.3 ± 12.2	13.8 ± 12.4	12.3 ± 11.4	11.6 ± 7.5	0.901
White blood cells, ×10^9^/L	9.3 ± 1.7	9.4 ± 2.7	9.4 ± 2.0	9.5 ± 2.3	0.990
Time from stent to cell therapy, hours	–	1.6 ± 0.9 h	4.7 ± 1.3 d	11.1 ± 3.3d	–
TIMI flow grade before study therapy	2.76 ± 0.44	2.89 ± 0.32	2.73 ± 0.45	2.81 ± 0.40	0.513
TIMI flow grade after study therapy	2.88 ± 0.33	2.93 ± 0.37	2.80 ± 0.40	2.88 ± 0.33	0.637
Number of BMC injected, ×10^8^	–	4.8 ± 2.5	5.0 ± 3.8	4.8 ± 1.8	–
CD34+, ×10^6^	–	1.8 ± 1.0	1.2 ± 0.8	0.9 ± 0.6	–
CD133+, ×10^5^	–	4.1 ± 2.7	3.0 ± 2.0	2.3 ± 2.1	–
Baseline ejection fraction (echocardiography), %	43.5 ± 3.5	44.7 ± 3.9	43.1 ± 6.0	43.1 ± 6.4	0.603
End-diastolic volume, ml	157.7 ± 26.1	153.1 ± 27.9	151.7 ± 21.8	154.5 ± 26.7	0.639
End-systolic volume, ml	93.9 ± 17.3	90.8 ± 19.3	90.5 ± 18.3	97.0 ± 25.1	0.523
Medication at discharge					
Aspirin (%)	100	96.1	100	100	0.420
Clopidogrel (%)	96.0	100	100	100	0.372
ACE inhibitor or ATII blocker, %	100	100	96.2	100	0.396
Beta-blocker, %	92.0	100	100	96.1	0.558
Statin, %	100	100	100	100	1.000
Medication at 12 months					
Aspirin, %	96.0	96.2	100	100	0.573
Clopidogrel, %	88.0	77.8	80.8	88.5	0.435
ACE inhibitor or ATII blocker, %	96.0	92.6	92.0	96.1	0.894
Beta-blocker, %	96.0	92.3	100	96.1	0.589
Statin, %	96.0	92.3	96.1	96.1	0.914

*ACE* angiotensin converting enzyme; *AMI* acute myocardial infarction; *ATII* Angiotensin receptor inhibitor; *CAD* coronary artery disease; *CON* control group; *CPR* cardiopulmonary resuscitation; *CRP* C-reactive protein; *group A* bone marrow mononuclear cell (BMC) infusion within 1 day after percutaneous coronary intervention (PCI); *group B* BMC infusion at 3–7 days after PCI; *group C* BMC infusion at 7–30 days after PCI; *LAD* left anterior descending; *LCX* Left cyclotron; *PCI* percutaneous coronary intervention; *RCA* right coronary artery; *TIMI* thrombolysis in myocardial infarction

### Clinical outcomes during 12 months of follow-up

As shown in Fig. 1, of the 110 AMI patients, 104 were enrolled; 84 had coronary arteriography at the 6-month follow-up, and 85 completed 12 months of follow-up.

The peak creatinine kinase (CK)-MB and cardiac troponin T (cTnT) levels and the time to peak for both markers were not significantly different among the four groups (*p* > 0.05). No significant differences in serum high-sensitive C-reactive protein (hsCRP) and CK-MB peak values before or after the operation were detected among the four groups. These results collectively suggest that no inflammation or new myocardial lesions occurred after cell transplantation.

No significant differences in the frequency of atrial premature beats, ventricular extrasystole, atrial tachycardia, and ventricular tachycardia on Holter monitoring were observed among the four groups during hospitalization. No proarrhythmic effects were detected on Holter monitoring during follow-up. During follow-up, there were no cases of death, tumor, or malignant arrhythmias. Compared to the control group, the occurrence of the combined clinical endpoint of death, MI recurrence, and rehospitalization due to heart failure tended to be lower in group A (*p* = 0.078) and group B (*p* = 0.214), but not in group C (*p* = 0.673). There was no significant difference in restenosis among the four groups (*p* > 0.05) (as shown in Table 2).

**Table 2:** Clinical events

	CON	Group A	Group B	Group C	*p* value
	(n = 25)	(n = 27)	(n = 26)	(n = 26)	
In-hospital course					
Death, n	0	0	0	0	1.000
MI relapse, n	0	0	0	0	1.000
Angina pectoris attack, n	3	1	2	2	0.747
Malignant arrhythmia, n	0	0	0	0	1.000
Fever (body temperature >37.5 °C) lasting at least 1 week	1	2	0	1	0.589
In-stent thrombus re-occlusion, n					
Drug eluting stent	0	1	0	1	0.590
Bare stent	1	0	0	0	0.372
12 months follow-up					
Death, n	0	0	0	0	1.000
MI relapse, n	2	1	0	1	0.574
Angina pectoris attack, n	2	1	1	3	0.773
Malignant arrhythmia, n	0	0	0	0	1.000
In-stent restenosis, n					
Drug eluting stent	1	1	0	1	0.894
Bare stent	1	0	1	2	0.589
Neoplasm, n	0	0	0	0	1.000
Revascularization, n	2	2	1	1	0.875
Rehospitalization due to heart failure, n	4	0	2	2	0.234
Others, n	0	0	0	0	1.000
Combined events (death, recurrence of myocardial infarction and rehospitalization for heart failure)	6	1	2	3	0.254

*CON* control group; *group A* bone marrow mononuclear cell (BMC) infusion within 1 day after percutaneous coronary intervention (PCI); *group B* BMC infusion at 3–7 days after PCI; *group C* BMC infusion at 7–30 days after PCI; *MI* myocardial infarct

### Quantitative variables of left ventricular function

**Fig. 2:**
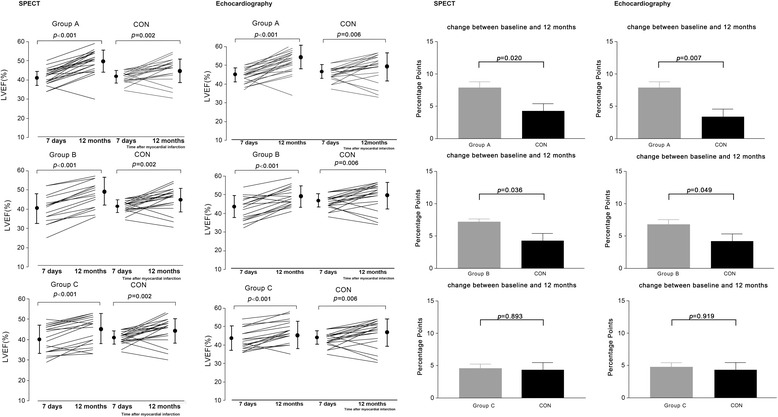
Left ventricular ejection fraction at baseline and at 12 months after myocardial infarction. Left ventricular ejection fraction (*LVEF*) determined by echocardiography initially and at 12-month follow-up in the four groups. Compared with baseline, global LVEF in the four groups was significantly increased on echocardiography at 12 months. Compared with the control group (*CON*), the absolute change in LVEF from baseline to 12 months was significantly higher in groups A and B, but not in group C. *group A* bone marrow mononuclear cell (BMC) infusion within 1 day after percutaneous coronary intervention (PCI); *group B* BMC infusion at 3–7 days after PCI; *group C* BMC infusion at 7–30 days after PCI

Baseline recordings were obtained for SPECT and echocardiography at 2.1 ± 0.8 and 3.2 ± 0.6 days, respectively. Baseline measurements of left ventricular function, volumes, and myocardial perfusion did not differ significantly among the four groups (Table 1). Compared with baseline (Table 3), global LVEF in the four groups was significantly increased on echocardiography at the 6-month follow-up (44.7 ± 3.9 % to 50.4 ± 4.7 % in group A, *p* < 0.001; 43.1 ± 6.0 % to 48.2 ± 6.4 % in group B, *p* < 0.001; 43.1 ± 6.4 % to 46.8 ± 6.5 % in group C, *p* < 0.001; 43.5 ± 3.5 % to 45.9 ± 5.4 % in CON, *p* < 0.001), and it was further improved at 12 months (52.2 ± 5.8 % in group A; 49.7 ± 5.6 % in group B; 47.4 ± 6.1 % in group C; 47.0 ± 6.9 % in CON). Compared to the control group (Fig. 2), the absolute change in LVEF from baseline to 12 months was significantly higher in groups A and B (*p* = 0.007 for group A vs CON and *p* = 0.049 for B vs CON), but not in group C (*p* = 0.919). Notably, the improvement in LVEF was similar between groups A and B (7.9 ± 4.9 % vs 6.9 ± 3.9 %, *p* = 0.455), and it was more significant in groups A and B than in group C (*p* < 0.01). Moreover, the decrease in LVESV from baseline to 12 months did not differ between groups A and B (*p* = 0.656) and between the CON group and group C (*p* = 0.468). However, the LVESV decrease was greater in groups A and B than in the CON group or group C (*p* < 0.05). By contrast, there was no significant difference among the four groups in the LVEDV decrease (*p* = 0.284) from baseline to the 12-month follow-up (Table 4). In addition, the 201Ti-SPECT data obtained at 12 months showed that myocardial perfusion was significantly enhanced in all four groups (*p* < 0.05) (Table 3). However, there was no significant difference in myocardial perfusion between groups A and B (6 months: *p* = 0.482; 12 months: *p* = 0.761) and between the CON group and group C (6 months: *p* = 0.838; 12 months: *p* = 0.862). Taken together, these results suggest that BMC transplantation within 24 hours or at 3–7 days after PCI further improves cardiac function in addition to the benefits derived from PCI, whereas BMC infusion performed later (7–30 days after acute PCI) offers no additional benefit.

**Table 3:** Analysis of left ventricular ejection fraction and myocardial perfusion defected by echocardiogram and 201Ti- single photon emission computed tomography

	CON (n = 25)	Group A (n = 27)	Group B (n = 26)	Group C (n = 26)
	(95 % CI)	(95 % CI)	(95 % CI)	(95 % CI)
Left ventricular ejection fraction on echocardiography, %				
Baseline	43.5 ± 3.5	44.7 ± 3.9	43.1 ± 6.0	43.1 ± 6.4
	(42.0 to 44.9)	(43.2 to 46.3)	(40.7 to 45.5)	(40.5 to 45.7)
6 months	45.9 ± 5.4^*^	50.4 ± 4.7^*†‡^	48.2 ± 6.4^*^	46.8 ± 6.5^*^
	(43.5 to 48.3)	(48.5 to 52.2)	(45.2 to 51.2)	(43.8 to 49.7)
Change from baseline	2.4 ± 3.2	5.6 ± 3.3^†‡^	5.5 ± 2.2^†‡^	2.9 ± 2.8
	(1.0 to 3.8)	(4.3 to 6.9)	(4.4 to 6.5)	(1.7 to 4.2)
12 months	47.0 ± 6.9*	52.2 ± 5.8^*†‡^	49.7 ± 5.6*	47.4 ± 6.1*
	(43.9 to 50.1)	(49.9 to 54.6)	(46.9 to 52.5)	(44.4 to 50.5)
Change from baseline	3.4 ± 5.7	7.9 ± 4.9^†‡^	6.9 ± 3.9^†‡^	4.7 ± 3.7
	(1.0 to 6.0)	(6.0 to 9.8)	(5.0 to 8.8)	(2.9 to 6.5)
Myocardial perfusion defect on (single photon emission computed tomography), %				
Baseline	42.0 ± 2.6	42.0 ± 3.8	41.2 ± 7.1	42.4 ± 7.5
	(40.9 to 43.0)	(40.5 to 43.6)	(38.3 to 44.2)	(39.4 to 45.5)
6 months	38.2 ± 5.0^*^	36.4 ± 5.2^*^	36.7 ± 6.7^*^	39.7 ± 7.0^*^
	(35.9 to 40.5)	(34.3 to 38.5)	(33.5 to 39.8)	(36.7 to 42.8)
Change from baseline	−3.5 ± 4.5	−5.7 ± 3.1^†^	−5.1 ± 3.0	−3.4 ± 3.2
	(−5.6 to −1.5)	(−6.9 to −4.4)	(−6.5 to −3.7)	(−4.8 to −2.0)
12 months	37.8 ± 6.0^*^	34.4 ± 6.5^*^	33.4 ± 6.6^*^	37.9 ± 7.0^*^
	(35.0 to 40.5)	(31.7 to 37.2)	(30.0 to 36.8)	(34.6 to 41.1)
Change from baseline	−4.7 ± 5.7	−7.8 ± 4.5^†‡^	−7.5 ± 2.9^†‡^	−5.0 ± 4.0
	(−7.2 to −2.1)	(−9.6 to −5.9)	(−9.0 to −6.0)	(−6.8 to −3.2)

^*^ *p* < 0.05, vs baseline; ^†^ *p* < 0.05, vs control; ^‡^ *p* < 0.05, vs group C. *CON* control group; *group A* bone marrow mononuclear cell (BMC) infusion within 1 day after percutaneous coronary intervention (PCI); *group B* BMC infusion at 3–7 days after PCI; *group C* BMC infusion at 7–30 days after PCI

**Table 4:** LVEDV and LVESV derived from echocardiography analysis

	CON (n = 25)	Group A (n = 27)	Group B (n = 26)	Group C (n = 26)
	(95 % CI)	(95 % CI)	(95 % CI)	(95 % CI)
LVEDV by echocardiography, ml				
Baseline	157.7 ± 26.1	153.1 ± 27.9	151.7 ± 21.8	154.5 ± 26.7
	(148.0 to 167.3)	(141.8 to 165.4)	(142.9 to 160.5)	(143.7 to 165.3)
6 months	156.9 ± 27.2	151.2 ± 26.3	149.8 ± 23. 5	152.8 ± 24.7
	(144.8 to 168.9)	(139.3 to 162.0)	(138.6 to 158.1)	(142.1 to 163.5)
Change from baseline	−2.5 ± 14.5	−4.0 ± 11.3	−3.7 ± 12.5	−3.8 ± 15.4
	(−8.9 to 3.9)	(−10.5 to 1.8)	(−9.3 to 2.7)	(−10.4 to 2.9)
12 months	157.5 ± 27.6	149.4 ± 25.8^†^	148.5 ± 20.7^†^	150.2 ± 26.0
	(145.3 to 169.7)	(137.2 to 160.5)	(137.9 to 156.2)	(138.4 to 162.1)
Change from baseline	−3.9 ± 18.7	−7.9 ± 13.9	−6.6 ± 10.6	−7.6 ± 18.0
	(−12.2 to 4.4)	(−14.3 to −0.8)	(−11.5 to −1.4)	(−15.8 to 0.6)
LVESV by echocardiography, ml				
Baseline	93.9 ± 17.3	90.8 ± 19.3	90.5 ± 18.3	97.0 ± 25.1
	(88.9 to 100.9)	(83.0 to 98.6)	(83.2 to 97.9)	(86.8 to 107.1)
6 months	90.6 ± 21.4	77.2 ± 19.0^*†‡^	83.2 ± 14.7	92.2 ± 21.6
	(80.8 to 100.3)	(69.3 to 85.0)	(76.3 to 90.0)	(82.9 to 101.6)
Change from baseline	−4.0 ± 10.8	−13.4 ± 10.0^†‡^	−10.5 ± 11.9	−5.3 ± 12.0
	(−8.9 to 0.8)	(−17.6 to −9.3)	(−16.0 to −4.9)	(−10.5 to −0.2)
12 months	89.8 ± 22.3^*^	71.1 ± 19.8^*†‡^	73.4 ± 17.1^*†‡^	89.1 ± 22.6^*^
	(80.8 to 100.3)	(62.6 to 79.7)	(64.5 to 82.2)	(78.8 to 99.4)
Change from baseline	−6.4 ± 15.9	−20.5 ± 13.3^†‡^	−19.6 ± 11.1^†‡^	−9.4 ± 16.3
	(−13.3 to 0.4)	(−26.2 to −14.7)	(−25.3 to −13.9)	(−16.8 to −1.9)

^*^ *p* < 0.05, vs baseline; ^†^ *p* < 0.05, vs control; ^‡^ *p* < 0.05, vs group C. *CON* control group; *group A* bone marrow mononuclear cell (BMC) infusion within 1 day after percutaneous coronary intervention (PCI); *group B* BMC infusion at 3–7 days after PCI; *group C* BMC infusion at 7–30 days after PCI; *LVEDV* left ventricular end-diastolic volume; *LVESV* left ventricular end-systolic volume

## Discussion

The principal finding of our study is that intracoronary infusion of BMCs within 24 hours or at 3–7 days after emergency PCI is associated with a significant increase in the recovery of left ventricular contractile function and remolding in AMI patients; intracoronary infusion of BMC at 7–30 days after PCI had no significant effect.

Myocardial infarction leads to scar formation and a subsequent reduction in cardiac performance. Stem cell-based regeneration provides a new strategy for the treatment of AMI patients. In addition to determining the effects of stem cells on left ventricular function, the optimal time window for cell infusion was also assessed. Several recent trials have reported conflicting results with respect to the optimal timing of cell therapy [2–6, 12]. The REPAIR-AMI trial found that the largest benefit occurred when cells were injected 5 to 6 days after infarction [5]. In another trial, patients with STEMI who underwent intracoronary injection of autologous BMCs on day 7 or after 6 months showed significant improvements in two-dimensional systolic strain in all segments and in the infarcted area only in the BMC group [13]. Janssens et al. reported no functional benefit derived from the injection of cells within the first 24 hours after infarction [14]. On the other hand, in our previous study with a small sample in which we followed patients for 3 months, emergency intracoronary administration of BMCs within 3 hours after primary PCI was found to be safe and practical [5]. Intracoronary transfer of autologous BMCs in patients with a healed myocardial infarct (13 ± 8 months) did not lead to a significant improvement of cardiac systolic function, infarct size or myocardial perfusion [15].

The optimal time frame for intracoronary cell therapy is a complex issue. It is probably determined by the equilibrium between factors that facilitate and those that inhibit the homing and cell survival that occurs during the post-myocardial infarction inflammatory process [16, 17]. Given the biological time course of healing and the expression of multiple factors, some researchers believe that the highest probability for optimal nesting and survival is in the period between day 3 and day 7 [18]. On the other hand, these factors are also related to stem cell homing to the infarct zone, which suggests that cell infusion in the early phase after myocardial infarction may be equally effective [19].

The optimal time of cell delivery has not been determined to date. Our meta-analysis showed that BMC transfer at 4 to 7 days post-AMI was the optimal time to improve cardiac function in AMI patients [20]. Therefore, in the present pilot study, we investigated the effect of BMC infusions at different times from 24 hours to 1 month after myocardial infarction. Our present study suggests that BMC infusion administered within 24 hours after the primary PCI is as effective as BMC infusion at 3 to 7 days after PCI. In the present study, BMC infusion after 7 days (11.1 ± 3.3 days) had no significant effects on the recovery of left ventricular function and remolding as compared to the control group; this finding was not consistent with the results reported by Fernández-Aviles et al., who injected mononuclear stem cells at an average of 2 weeks after infarction and reported a comparable increase in left ventricular function [21]. In another similar trial, patients with STEMI who underwent successful primary PCI and administration of intra-coronary BMCs at either 3 or 7 days following the event showed recovery of global and regional left ventricular function similar to that of placebo-treated patients [12]. The reasons for the different results are unclear. The study by Fernández-Aviles et al. included only a few patients and had no control group. The sample size in the TIME study was also small and the average LVEF of enrolled patients was >45 %. The TIME study also suggested that, in STEMI patients, myocardial repair was more dependent on baseline BMC characteristics (CD31+ BMC) than on whether the patient underwent intracoronary BMC transplantation [22].

In most clinical trials, the average baseline LVEF of AMI patients is approximately 50 % [23]. The subgroup outcome from REPAIR-AMI indicated that patients with an LVEF of <49 % received a greater benefit (7.5 % vs 5.5 %) from cell transplantation [24]. The magnitude of left ventricular contractile recovery appears to be inversely related to the baseline LVEF. Patients enrolled in our study had substantial functional impairment: on average, the global LVEF at baseline was 41 %, which is much lower than that reported by earlier studies. After successful myocardial reperfusion and autologous BMC transplantation, LVEF increased by 5–6 % at 6 months and by 7–8 % at 12 months. These results are similar to those of the subgroup with a lower baseline LVEF in REPAIR-AMI. In addition, preliminary studies have suggested that the number of cells transplanted plays a role in the clinical outcome [4, 5, 25, 26]. In our study, the marked beneficial effects of BMC infusion on the recovery of contractile function were likely the result of the larger number of cells delivered (6.9 ± 8.8 × 10^8^); this number was almost ten times higher than the number of cells delivered in BOOST or TOPCARE-AMI.

Compared to the control group, the incidence of individual adverse clinical effects were lower in groups A and B (*p* = 0.078 for the comparison with group A, and *p* = 0.214 for the comparison with group B). These results suggest that BMC infusion reduces the risk of chronic heart failure, which is a common complication of myocardial infarction. Importantly, we have not found that stem cell transplantation increases the rate of in-stent restenosis and increases the hsCRP or cTnT levels. Notably, there was no mortality at 12 months in any of the groups. This is a moderate-risk population. The average LVEF at baseline was 40.5 % to 46.3 %. The clinical characteristics of these patients were lower CRP peak levels and fewer three-vessel coronary artery lesions, which predicted better prognosis. Furthermore, almost all these patients took optimal medical treatments including aspirin, statins, beta-blockers and angiotensin converting enzyme inhibitors after PCI. Of course, the small number of patients and the limited follow-up time in this study might be related to the lower incidence of adverse effects in this study. Thus, further work is still needed on the safety of intracoronary BMC infusion despite our previous results [27, 28].

There are a number of SPECT measurements to assess myocardial perfusion defects, such as the two-day 201Tl-SPECT technique, 99mTc-MIBI-SPECT imaging and exercise SPECT. In the present study, we used a one-day 201Tl–SPECT technique because the baseline SPECT data were obtained within a few days after myocardial infarction. This was shown to be a safe and feasible method in the present study.

Our study had several limitations. First, although the control group received an intracoronary saline infusion at the same time point as group A, we did not have control groups receiving the infusion at the same times points as groups B and C (although saline infusion alone has no effect on AMI [1–6]). Second, we did not label the cells to estimate cell survival and homing rates, and such observations might provide explanations for the differences between our findings and those of other researchers. In view of findings showing increased rates of cell death associated with inflammation during the early stage of infarction, the REPAIR-AMI trial suggested that early cell infusions were less effective than those delayed beyond 5 days. However, we found early infusion to be as effective as infusion at 3–7 days. Our results might be partly attributable to our delivery of a large number of BMCs and the detection of a larger population of CD34-positive cells in the BMCs of patients within 1 day after AMI, compared to other studies. Additionally, we previously showed that myocardial stroma cell derived factor-1 (SDF-1) expression increased and peaked at the first day post-AMI in rats; stroma cell derived factor-1 expression is important for progenitor cell chemotaxis, homing, engraftment, and retention in the damaged myocardium, and BMC enrichment and angiogenesis in the host hearts were more abundant in the infarcted heart. Third, cardiac magnetic resonance imaging is considered the best modality to assess left ventricular remodeling and function after myocardial infacrtion. Although we measured the infarcted area by cardiac magnetic resonance imaging at 6 and 12 months in some patients, the data were not included in the present study because we did not perform baseline magnetic resonance imaging at 2–3 days in all patients. Fourth, the benefits in functional parameters failed to translate into clinically meaningful improvement in outcomes, and the combined endpoint of death, myocardial infarction, and rehospitalization for heart failure was not significantly different at 1 year between the groups. One explanation is that our sample size was too small. In addition, only 85 patients completed the 6- and 12-month follow-up periods. Owing to the small number of patients and the duration of follow-up, our study was not powered to assess the optimal timing. However, our results provide insight into the optimal timing and sufficient background for its assessment in a large-scale trial. Another possibility is that the benefits of BMC infusion are associated with the regeneration of the myocardium and vessels, and the volume of tissue regeneration at 1 year may be too small to compensate for the initial damage. Alternatively, the benefits may be dependent on the presence of cytokines and growth factors released from transplanted BMCs, which would have disappeared at 1 year. Repeated BMC infusion at a certain time after the initial therapy might improve the results [29].

## Conclusion

In summary, the primary outcomes showed that, in AMI patients, intracoronary BMC infusion within 24 hours after the primary PCI is as effective as BMC infusion 3 to 7 days after primary PCI with respect to left ventricular contractile function recovery and remodeling. Of course, it needs further data from more trials.

## Abbreviations

AMI: acute myocardial infarction
ANOVA: analysis of variance
BMC: bone marrow mononuclear cell
CK: creatinine kinase
CON: control group
cTnT: cardiac troponin T
hsCRP: high-sensitive C-reactive protein
IRA: infarct-related artery
LVEDV: left ventricular end-diastolic volume
LVEF: left ventricular ejection fraction
LVESV: left ventricular end-systolic volume
PCI: percutaneous coronary intervention
SPECT: single photon emission computed tomography
STEMI: ST-elevation myocardial infarction

## References

[1] Angeli FS, Caramori PR, da Costa Escobar Piccoli J, Danzmann LC, Magedanz E, Bertaso A (2012). Autologus transplantation of mononuclear bone marrow cells after acute myocardial infarction: a PILOT study. Int J Cardiol.

[2] Traverse JH, Henry TD, Pepine CJ, Willerson JT, Ellis SG (2014). One-year follow-up of intracoronary stem cell delivery on left ventricular function following ST-elevation myocardial infarction. JAMA.

[3] Delewi R, Hirsch A, Tijssen JG, Schächinger V, Wojakowski W, Roncalli J (2014). Impact of intracoronary bone marrow cell therapy on left ventricular function in the setting of ST-segment elevation myocardial infarction: a collaborative meta-analysis. Eur Heart J.

[4] Makkar RR, Smith RR, Cheng K, Malliaras K, Thomson LE, Berman D (2012). Intracoronary cardiosphere-derived cells for heart regeneration after myocardial infarction (CADUCEUS): a prospective, randomised phase 1 trial. Lancet.

[5] Schächinger V, Erbs S, Elsässer A, Haberbosch W, Hambrecht R, Hölschermann H (2006). REPAIR-AMI Investigators. Improved clinical outcome after intracoronary administration of bone-marrow-derived progenitor cells in acute myocardial infarction: final 1-year results of the REPAIR-AMI trial. Eur Heart J.

[6] Wöhrle J, von Scheidt F, Schauwecker P, Wiesneth M, Markovic S, Schrezenmeier H (2013). Impact of cell number and microvascular obstruction in patients with bone-marrow derived cell therapy: final results from the randomized, double-blind, placebo controlled intracoronary Stem Cell therapy in patients with Acute Myocardial Infarction (SCAMI) trial. Clin Res Cardiol.

[7] Ge J, Li Y, Qian J, Shi J, Wang Q, Niu Y (2006). Efficacy of Emergent Transcatheter Transplantation of Stem Cells for Treatment of Acute Myocardial Infarction (TCT-STAMI). Heart.

[8] Gyöngyösi M, Lang I, Dettke M, Beran G, Graf S, Sochor H (2009). Combined delivery approach of bone marrow mononuclear stem cells early and late after myocardial infarction: the MYSTAR prospective, randomized study. Nat Clin Pract Cardiovasc Med.

[9] Sürder D, Schwitter J, Moccetti T, Astori G, Rufibach K, Plein S (2010). Cell-based therapy for myocardial repair in patients with acute myocardial infarction: rationale and study design of the SWiss multicenter Intracoronary Stem cells Study in Acute Myocardial Infarction (SWISS-AMI). Am Heart J.

[10] Otterstad JE (2002). Measuring left ventricular volume and ejection fraction with the biplane Simpson’s method. Heart.

[11] Ho FM, Huang PJ, Liau CS, Lee FK, Chieng PU, Su CT (1995). Dobutamine stress echocardiography compared with dipyridamole thallium-201 single-photon emission computed tomography in detecting coronary artery disease. Eur Heart J.

[12] Traverse JH, Henry TD, Pepine CJ, Willerson JT, Zhao DX, Ellis SG (2012). Cardiovascular Cell Therapy Research Network (CCTRN), effect of the use and timing of bone marrow mononuclear cell delivery on left ventricular function after acute myocardial infarction: the TIME randomized trial. JAMA.

[13] Plewka M, Krzemińska-Pakuła M, Lipiec P, Peruga JZ, Jezewski T, Kidawa M (2009). Effect of intracoronary injection of mononuclear bone marrow stem cells on left ventricular function in patients with acute myocardial infarction. Am J Cardiol.

[14] Janssens S, Dubois C, Bogaert J, Theunissen K, Deroose C, Desmet M (2006). Autologous bone marrow-derived stem-cell transfer in patients with ST-segment elevation myocardial infarction: double-blind, randomized controlled trial. Lancet.

[15] Yao K, Huang R, Qian J, Cui J, Ge L, Li Y (2008). Administration of intracoronary bone marrow mononuclear cells on chronic myocardial infarction improves diastolic function. Heart.

[16] Frangogiannis NG, Smith CW, Entman ML (2002). The inflammatory response in myocardial infarction. Cardiovasc Res.

[17] Saparov A, Chen CW, Beckman SA, Wang Y, Huard J (2013). The role of antioxidation and immunomodulation in postnatal multipotent stem cell-mediated cardiac repair. Int J Mol Sci.

[18] Deten A, Volz HC, Briest W, Zimmer HG (2002). Cardiac cytokine expression is upregulated in the acute phase after myocardial infarction. Experimental studies in rats. Cardiovasc Res.

[19] Ma J, Ge J, Zhang S, Sun A, Shen J, Chen L (2005). Time course of myocardial stromal cell-derived factor 1 expression and beneficial effects of intravenously administered bone marrow stem cells in rats with experimental myocardial infarction. Basic Res Cardial.

[20] Zhang S, Sun A, Xu D, Yao K, Huang Z, Jin H (2009). Impact of timing on efficacy and safety of intracoronary autologous bone marrow stem cells transplantation in acute myocardial infarction: a pooled subgroup analysis of randomized controlled trials. Clin Cardiol.

[21] Fernández-Aviles F, San Román JA, García-Frade J, Fernández ME, Peñarrubia MJ, de la Fuente L (2004). Experimental and clinical regenerative capability of human bone marrow cells after myocardial infarction. Circ Res.

[22] Schutt RC, Trachtenberg B, Cooke JP, Traverse JH, Henry TD, Pepine CJ, et al. Bone marrow characteristics associated with changes in infarct size after STEMI: a biorepository evaluation from the CCTRN TIME Trial. Circ Res. 2015;116(1):99–107. 10.1161/CIRCRESAHA.116.304710 PMC4282599 25406300

[23] Traverse JH, Henry TD, Vaughan DE, Ellis SG, Pepine CJ, Willerson JT (2010). Cardiovascular Cell Therapy Research Network. LateTIME: a phase-II, randomized, double-blinded, placebo-controlled, pilot trial evaluating the safety and effect of administration of bone marrow mononuclear cells 2 to 3 weeks after acute myocardial infarction. Tex Heart Inst J.

[24] Dill T, Schächinger V, Rolf A, Möllmann S, Thiele H, Tillmanns H (2009). Intracoronary administration of bone marrow-derived progenitor cells improves left ventricular function in patients at risk for adverse remodeling after acute ST-segment elevation myocardial infarction: results of the Reinfusion of Enriched Progenitor cells And Infarct Remodeling in Acute Myocardial Infarction study (REPAIR-AMI) cardiac magnetic resonance imaging substudy. Am Heart J.

[25] Gao LR, Pei XT, Ding QA, Chen Y, Zhang NK, Chen HY (2013). A critical challenge: dosage-related efficacy and acute complication intracoronary injection of autologous bone marrow mesenchymal stem cell in acute myocardial infarction. Int J Cardiol.

[26] Tambara K, Sakakibara Y, Sakaguchi G, Lu F, Premaratne GU, Lin X (2003). Transplanted skeletal myoblasts can fully replace the infracted myocardium when they survive in the host in large numbers. Circulation.

[27] Yao K, Huang RC, Ge L, Qian JY, Li YL, Xu SK, et al. Observation on the safety: clinical trial on intracoronary autologous bone marrow mononuclear cells transplantation for acute myocardial infarction. Zhong Hua Xin Xue Guan Bing Za Zhi (Chin). 2006;34:577–81. 17081355

[28] Rongchong H. Stem cell therapy for the treatment of coronary heart disease: safety evaluation. Zhong Hua Xin Xue Guan Bing Za Zhi (Chin). 2012;40:1–2. 23141081

[29] Yao K, Huang R, Sun A, Qian J, Liu X, Ge L (2009). Repeated autologous bone marrow mononuclear cell therapy in patients with large myocardial infarction. Eur J Heart Fail.

